# Bonding-Based Wafer-Level Vacuum Packaging Using Atomic Hydrogen Pre-Treated Cu Bonding Frames

**DOI:** 10.3390/mi9040181

**Published:** 2018-04-13

**Authors:** Koki Tanaka, Hideki Hirano, Masafumi Kumano, Joerg Froemel, Shuji Tanaka

**Affiliations:** 1 Department of Robotics, Tohoku University, 6-6-01, Aza Aoba, Aramaki Aoba-ku, Sendai 980-8579, Japan; koki.tanaka@tel.com (K.T.); tanaka@mems.mech.tohoku.ac.jp (S.T.); 2 Micro System Integration Center, Tohoku University, 6-6-01, Aza Aoba, Aramaki Aoba-ku, Sendai 980-8579, Japan; kumano@mems.mech.tohoku.ac.jp; 3 WPI-Advanced Institute for Materials Research, Tohoku University, 2-1-1 Katahira, Aoba-ku, Sendai 980-8577, Japan; joerg.froemel.e5@tohoku.ac.jp

**Keywords:** wafer bonding, wafer-level vacuum packaging, Cu thermos-compression bonding, atomic hydrogen, hot wire, nano-grain

## Abstract

A novel surface activation technology for Cu-Cu bonding-based wafer-level vacuum packaging using hot-wire-generated atomic hydrogen treatment was developed. Vacuum sealing temperature at 300 °C was achieved by atomic hydrogen pre-treatment for Cu native oxide reduction, while 350 °C was needed by the conventional wet chemical oxide reduction procedure. A remote-type hot-wire tool was employed to minimize substrate overheating by thermal emission from the hot-wire. The maximum substrate temperature during the pre-treatment is lower than the temperature of Cu nano-grain re-crystallization, which enhances Cu atomic diffusion during the bonding process. Even after 24 h wafer storage in atmospheric conditions after atomic hydrogen irradiation, low-temperature vacuum sealing was achieved because surface hydrogen species grown by the atomic hydrogen treatment suppressed re-oxidation. Vacuum sealing yield, pressure in the sealed cavity and bonding shear strength by atomic hydrogen pre-treated Cu-Cu bonding are 90%, 5 kPa and 100 MPa, respectively, which are equivalent to conventional Cu-Cu bonding at higher temperature. Leak rate of the bonded device is less than 10^−14^ Pa m^3^ s^−1^ order, which is applicable for practical use. The developed technology can contribute to low-temperature hermetic packaging.

## 1. Introduction

Hermetic packaging is a key technology for MEMS (micro electro mechanical systems) sensors which are applied to mobile devices, cars, and environment measurements in terms of protection of MEMS structures and materials from environmental particles, gases, and humidity to ensure reliability and stability. Moreover, high vacuum sealing is required for inertial sensors such as gyroscopes and resonators to reduce air dumping and for IR (infra-red) sensors to keep thermal isolation [1]. For these applications, wafer-level vacuum packaging using wafer bonding technology has been developed [2]. The bonding technology is also useful for the integration of MEMS with heterogeneous elements such as CMOS (complementary metal-oxide semiconductor) to obtain adequate performance [3]. Many wafer-bonding techniques for wafer-level hermetic packaging have been developed such as silicon fusion bonding [4], anodic bonding [5,6] and frit-glass bonding [7]. Silicon fusion bonding is not suitable for integrating MEMS with CMOS due to its high process temperature. Anodic bonding has risks of alkali ion contamination and high voltage damage to COMS. Historically, frit-glass bonding technology has been widely used for hermetic sealing, but a bonding temperature above 400 °C can damage modern CMOS, which has a limited thermal budget. Moreover, a frit-glass bonding frame wider than 100 µm is a serious cost factor for devices with shrinking die size. Solid-liquid inter-diffusion (SLID) bonding based on intermetallic compounds has emerged for high-temperature applications due to its ability to withstand higher temperatures than the bonding temperature [8]. However, the presence of voids at the bonding frame due to atomic diffusion (Kirkendall voids) can cause a reliability risk. Process instability due to the formation of intermetallic compounds during storage is also a problem that is difficult to control.

On the other hand, metal thermo-compression bonding is a promising technology for wafer-level integration and packaging, in terms of vacuum sealing possibility using narrow bonding frames together with electrical interconnection. In particular, Au is a suitable material for low-temperature bonding, because Au as a noble metal is stable in terms of oxidation. Hence, vacuum sealing by Au-Au bonding can be performed at 300 °C without any pre-treatments such as surface oxide removal [9]. However, Au has not been commonly used as bonding material due to high cost and serious risk for contamination of semiconductor fabrication lines. On the other hand, Cu is known as a low-cost material and is widely used in the back-end process of semiconductor manufacturing. However, low-temperature vacuum sealing by thermo-compression bonding using Cu frames is difficult, because the native oxide layer on the Cu surface, which cannot be removed below 350 °C, prevents Cu atomic diffusion for achieving strong bonding strength [10,11]. Thus, Cu surface pre-treatment to remove the native oxide layer must be employed for low-temperature vacuum bonding. A highly reliable bonding interface was obtained by surface-activated Cu-Cu bonding (SAB) even at room temperature [12]. However, SAB technology requires an expensive tool, which enables wafer bonding followed by native oxide removal by Ar ion beam irradiation in the same vacuum chamber. Recently, surface-modified bonding using Cu nanoparticles was proposed. Practical bonding strength was obtained at 200 °C, but hermetic sealing has not been achieved [13]. In addition, a pre-treatment process should be implemented at lower than Cu re-crystallization temperature, because the coalescence of Cu fine grains during the bonding process plays an important role in filling interfacial gaps and voids which cause air leaking [14,15]. Typical Cu re-crystallization temperature is reported as the range of 120–150 °C [16].

Conventionally, wet pre-treatment methods, such as immersion into citric acid solution, have been used to remove Cu surface oxides; however, the treated Cu surface is easily re-oxidized when it is exposed in the atmosphere. To prevent the re-oxidization, special wafer bonding tools having in situ oxide removal methods such as formic acid vapor treatment have been developed [17]. However, oxide removal by formic acid vapor requires temperatures above 150 °C which starts the recrystallization of Cu grain. The corrosion of some metals such as Ti and Al by formic acid is also a problem. The protection of Cu surface from re-oxidation in the air by a self-assembled monolayer (SAM) has been proposed [18]. The process is simple, but wet SAM treatment can damage MEMS structures. In addition, SAM must be removed before bonding by heating at 200 °C, which exceeds Cu re-crystallization temperature [19].

As an alternative technique for oxide removal, H_2_ plasma pre-treatment was proposed [20,21]. Atomic hydrogen generated by H_2_ plasma cannot only reduce existing Cu oxides, but also form a Cu hydride-like layer, which prevents re-oxidation at the same time. Even though the Cu bonding frames were exposed to the air after H_2_/Ar plasma pre-treatment, sufficient bond strength for vacuum sealing at 300 °C was demonstrated [22]. Metal corrosion can be avoided by H_2_ plasma pretreatment. However, sufficient reduction of native oxide on Cu was not achieved below 150 °C [21].

To lower the pre-treatment temperature, atomic hydrogen generated by the catalytic decomposition of molecular hydrogen (H_2_) on the heated metal wire surface, i.e., “hot wire” has been developed. Izumi reported that Cu oxide can be reduced using atomic hydrogen by a hot wire even at 40 °C of substrate temperature [23]. It is noteworthy that density of atomic hydrogen by the hot-wire method is 1–2 orders of magnitude greater than that in plasma [24]. However, conventional open-type hot-wire reactors, wherein a hot wire and a sample are in the same chamber with a few centimeters of gap, heat up the sample to 150–200 °C by radiation from the hot wire [25]. In addition, thermally isolated thin structures, such as cantilevers and membranes, will be heated up higher than damaging temperature, e.g., 250–300 °C [25]. To reduce heating-up from radiation, a remote-type hot-wire tool, wherein atomic hydrogen is generated in a glass chamber, and conveyed to another chamber containing the substrate across a distance of 100–200 mm, has been developed [26]. Using this setup, oxide removal lower than Cu recrystallization temperature (120 °C) is expected. The proposed low-temperature pretreatment by the remote-type hot-wire tool is incompatible with high-temperature degassing procedure, which is commonly used for high-vacuum MEMS packaging for e.g., uncooled infrared microbolometer and MEMS gyroscope. On the other hand, a thin film getter is available to improve vacuum level in the package, because the getter is not activated during the low-temperature hot-wire pretreatment.

In this study, a thermo-compression bonding process of atomic hydrogen-pretreated Cu sealing frames utilizing a remote-type hot-wire tool was demonstrated. Re-oxidation suppression effect of the atomic hydrogen-treated Cu film and its surface conditions were investigated by AES (Auger electron spectroscopy) and TDS (thermal desorption spectroscopy) measurements.

## 2. Experimental

### 2.1. Remote-Type Hot-Wire Tool

**Figure 1:**
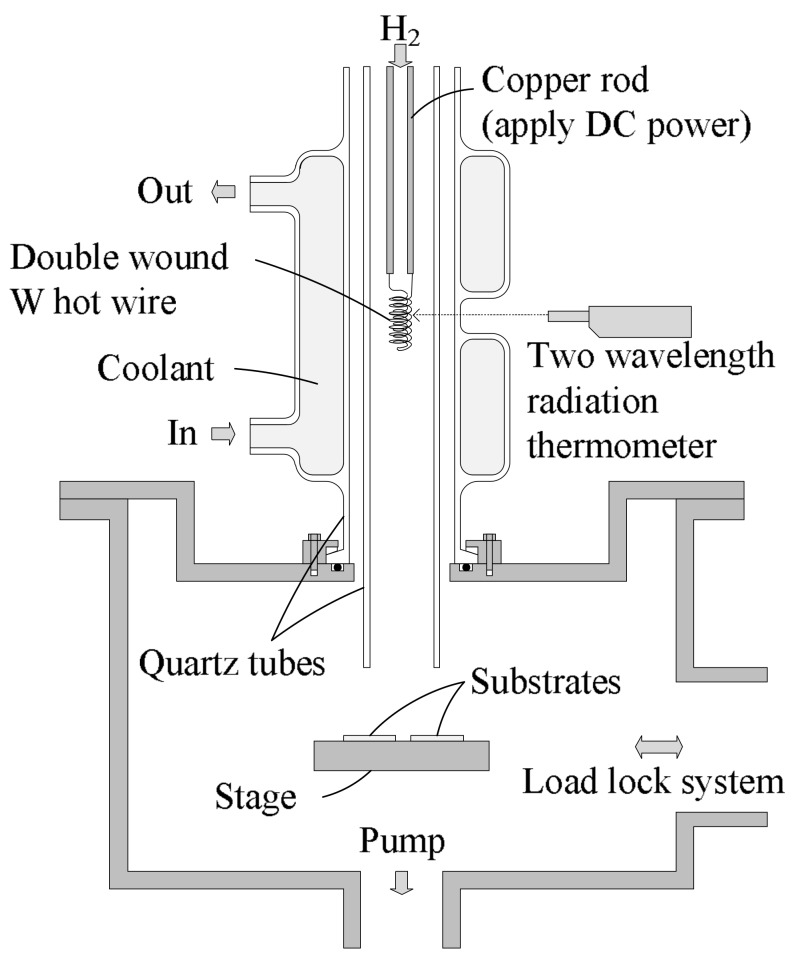
Schematic of the remote-type hot-wire tool.

Figure 1 shows schematic of a remote-type hot-wire tool as atomic hydrogen generator. Introduced H_2_ gas is dissociated to atomic hydrogen on the electrical heated tungsten wire surface by catalytic reaction. The tungsten wire inside a quartz tube is double wound (wire diameter: 0.5 mm, length: 450 mm, inside/outside winding diameter: 4/9 mm) to prevent thermal deformation. Generated atomic hydrogen at the hot wire is carried to the substrates along with high-speed viscous H_2_ flow. Because the annihilation of atomic hydrogen in the carrier gas (H_2_) is suppressed by Reaction (1),

H_2_ + H• → H• + H_2_, (1)

and the deactivation rate of atomic hydrogen on quartz surface is much lower than that on metals, a sample substrate can be treated with high atomic hydrogen density. The hot-wire temperature reaches 2100 °C measured by a two-wavelength radiation thermometer. However, the wafer substrate temperature during atomic hydrogen irradiation is lower than 100 °C, because thermal radiation from the hot wire is reduced by keeping a proper distance between the hot wire and the wafer. In this setup, the solid angle of the hot wire from the substrate is sufficiently small (~0.0023 sr), and the amount of the radiation to the substrates is negligibly small. Thus, the substrate stage temperature and hot-wire temperature can be controlled independently.

Atomic hydrogen density on the substrates evaluated by transmittance change of WO_3_ doped phosphate glass [27] is approximately 5.0 × 10^12^ cm^−3^, which is one order of magnitude higher than that in plasma [24]. H_2_ pressure in the chamber is controlled at 130 Pa by 500 sccm of H_2_ flow rate under evacuation by a mechanical booster pump.

### 2.2. Bonding Sample Preparation

**Figure 2:**
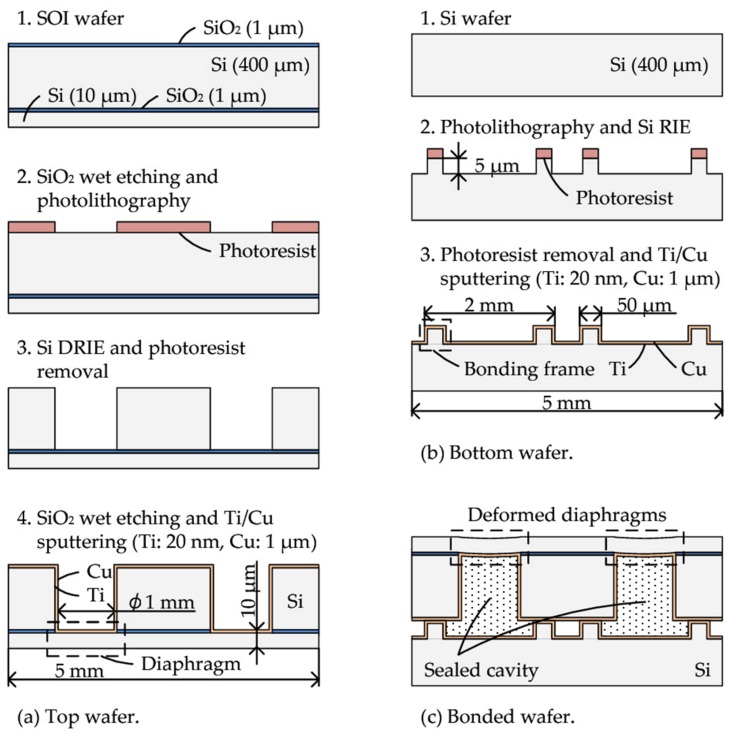
Fabrication processes of (**a**) the top wafer (cavity wafer), (**b**) the bottom wafer (bonding frame wafer) and (**c**) bonded wafer. Both wafer size is 2 cm square. Each wafer has 16 cavities with diaphragms and bonding frames.

Cu-Cu bonding is carried out by using a pair of 2 cm square sample wafers, because physics and the principle of bonding procedure for a 2 cm square wafer is essentially the same as larger wafers, and the developed bonding technology can be scalable to larger wafers. The fabrication processes are shown in Figure 2. A top wafer (cavity wafer) has cavities, and 10 µm thick Si diaphragms made by DRIE (deep reactive ion etching) of a silicon-on-insulator (SOI) wafer with a 10 µm-thick device layer followed by wet HF etching of a 1 µm-thick buried oxide (BOX) layer (Figure 2a). Each completed 2 cm square wafer has 36 diaphragms with 1 mm diameter. A bottom wafer (bonding frame wafer) has 10 µm high and 50 µm wide bonding frame structures made by Si DRIE (Figure 2b). On both wafers, 20 nm of Ti as adhesion layer and 1 µm of Cu layers as bonding layer were deposited by RF magnetron sputtering. Figure 2c shows completed samples after wafer bonding.

### 2.3. Pre-Treatment and Wafer Bonding

Citric acid pre-treatment and atomic hydrogen pre-treatment conditions are shown in Table 1. The top wafer and the bottom wafer are pre-treated at the same time and intentionally exposed to the atmosphere at 25 °C for 1 h or 24 h under 50% of relative humidity. The pair of wafers are introduced into a lab-made bonding system [28]. After bonding chamber pressure has reached 1.0 × 10^−2^ Pa by a turbo molecular pump, the wafers are aligned and transferred to the bonding stage at pre-set bonding temperature. Then the wafers are bonded by 70 MPa of bonding pressure. A graphite sheet is inserted between the bottom wafer and the bonding stage to uniformize bonding pressure distribution. The bonding conditions are shown in Table 2.

### 2.4. Evaluation of Bonding Performance

**Figure 3:**
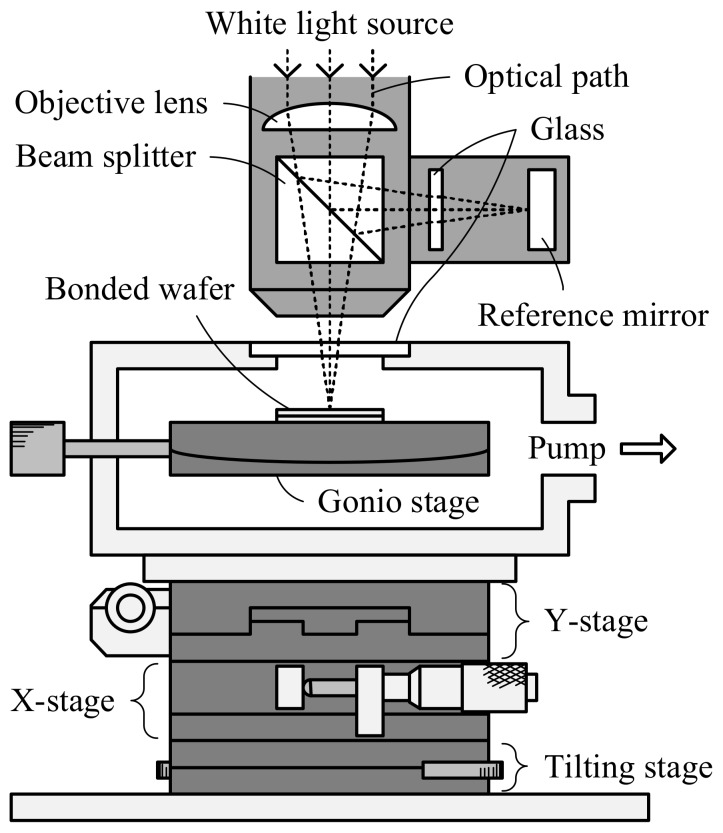
Schematic of the sealed cavity pressure evaluation by zero-balance method.

The accomplishment of vacuum sealing is judged by the deflection of the diaphragm due to differential pressure between the inside and outside of the cavity. Vacuum sealing yield is defined by dividing the number of deflected diaphragms by the total number of diaphragms. The actual pressure in the sealed cavity is evaluated by the 10 μm-thick diaphragm deformation in a pressure-controllable vacuum chamber at different pressures at 25 °C. The pressure inside the cavity is identical to the chamber pressure when the diaphragm is flat; this is called zero-balance method [9,29,30]. The deformation of the diaphragm is measured by a white light interferometry surface topography measurement system (MSA-500 Micro System Analyzer, Polytec GmbH, Waldbronn, Germany) through a glass window on the chamber, as shown in Figure 3. The leak rate of the hermetic sealed devices is evaluated by cavity pressure change with storage time in the air at 25 °C.

**Figure 4:**
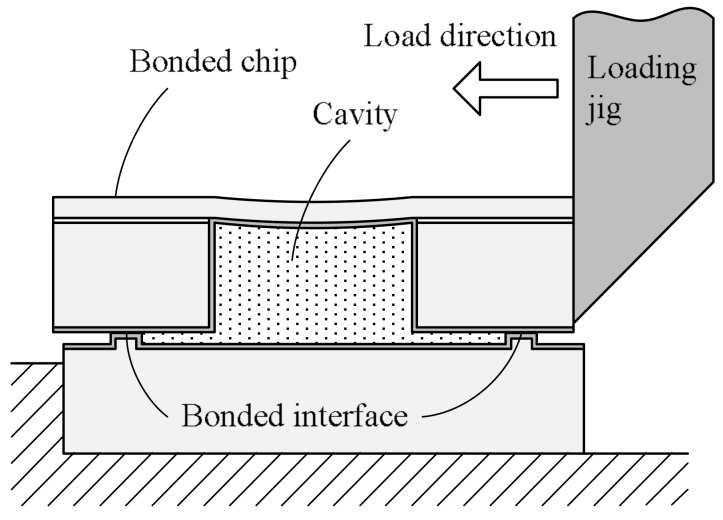
Schematic of bonding shear strength evaluation.

The bonding strength of the bonded wafers is evaluated using a shear tester (PTR-1101, Rhesca, Tokyo, Japan) at 25 °C. The schematic of the shear test setup is shown in Figure 4. The bonded wafers are diced into 2.5 mm square chips which have a single bonding frame. Shear force is applied to the bonded chip in the horizontal direction using the loading jig.

### 2.5. AES and TDS Measurements

Cu surface compositions change after the treatment by citric acid or atomic hydrogen is evaluated using X-ray induced AES measurement. A Mg-Kα X-ray source is used to separate the AES peaks of Cu_2_O and Cu.

Desorbed gas species from the treated Cu surface were identified by TDS method (TDS1200, ESCO, Ltd., Tokyo, Japan) at temperatures ranging from 50 to 500 °C, which simulates the bonding procedure. The heating rate is 1 K/s. [31,32]. The analysis of the TDS measurement system is quantitatively calibrated by hydrogen-implanted silicon samples.

The sample preparation and pre-treatment condition for the AES and TDS measurements are the same as the bonding experiment as shown in Table 1.

## 3. Results

### 3.1. Yield of Hermetic Sealing

**Figure 5:**
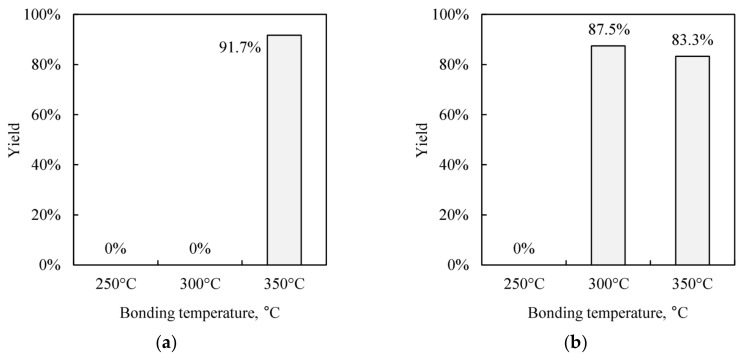
Yields of the hermetic sealed cavities. A pair of bonding wafers is exposed to the atmosphere for 1 h after each pre-treatment. (**a**) Citric acid treated wafer. (**b**) Atomic hydrogen treated wafer.

Figure 5a,b show the hermetic sealing yield of citric acid pre-treated wafer and the atomic hydrogen pre-treated wafer, respectively. Both wafers are intentionally exposed in the air for 1 h before bonding. The citric acid pre-treated wafers require bonding temperature higher than 350 °C for hermetic sealing. In contrast, hermetic Cu-Cu bonding was successfully demonstrated as low as 300 °C after atomic hydrogen pre-treatment. Even after exposing the wafers for 24 h in air after pre-treatment by atomic hydrogen, hermetic sealing was achieved. Therefore, atomic hydrogen treatment can improve hermetic sealing tolerance against air exposure and greatly relax the time constraint between the pretreatment and bonding process.

### 3.2. Bonding Strength

**Figure 6:**
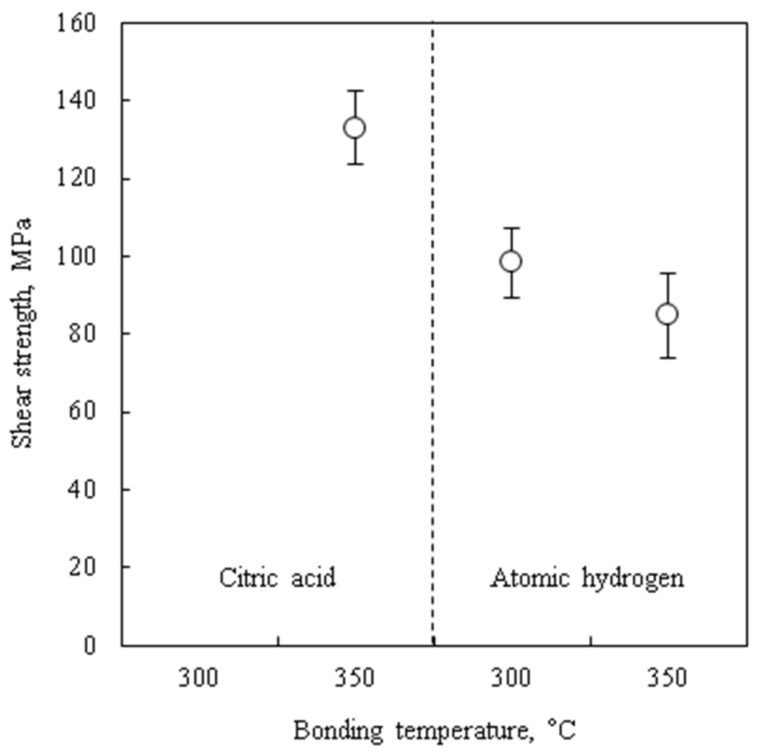
Shear strengths of the bonded chips.

Figure 6 shows the shear strengths of the bonded chips in different conditions. The shear strengths of the citric acid pre-treated wafer bonded at 300 °C is not evaluated because of zero hermetic sealing yield. The shear strength of the citric acid pre-treated chips and the atomic hydrogen pre-treated chips are 130 MPa and a range between 80 and 100 MPa, respectively. The shear strength of the atomic hydrogen pre-treated chips is lower than that of the citric acid pre-treated chips. However, it can be applied for actual use because the strength is sufficiently higher than MIL-STD-883G requirement (12.6 MPa) for the packaged devices [33].

### 3.3. Pressure of Sealed Cavity

**Figure 7:**
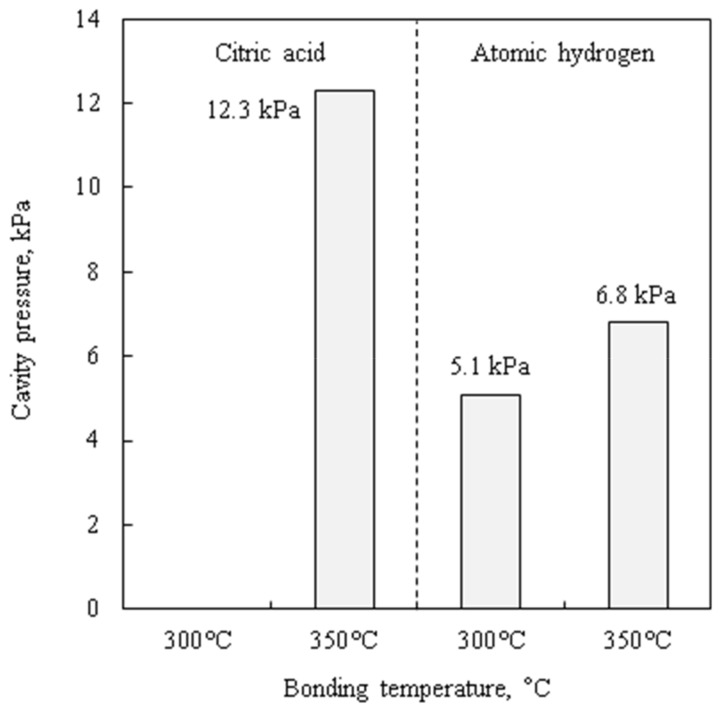
Hermetic sealed cavities pressure measured by zero-balance method.

Figure 7 shows sealed cavity pressures measured by the zero-balance method. The cavity pressure of the citric acid pre-treated wafer bonded at 300 °C cannot be evaluated because no sealed cavity is obtained. The cavity pressure is larger than 5 kPa, even though the bonding is performed under 1.0 × 10^−2^ Pa. The remaining cavity pressure is considered to be thermally desorbed gas from adsorbed molecules on the Cu films by the result of TDS measurements. The atomic hydrogen-treated wafers have approximately half the pressure of the citric acid-treated one. It is considered that atomic hydrogen reacts and decomposes adsorbed species on the Cu films. Bonding temperature lower than gas desorption temperature can reduce cavity pressure. The usage of getter materials for residual gas adsorption can also improve vacuum level.

### 3.4. Leak Rate

**Figure 8:**
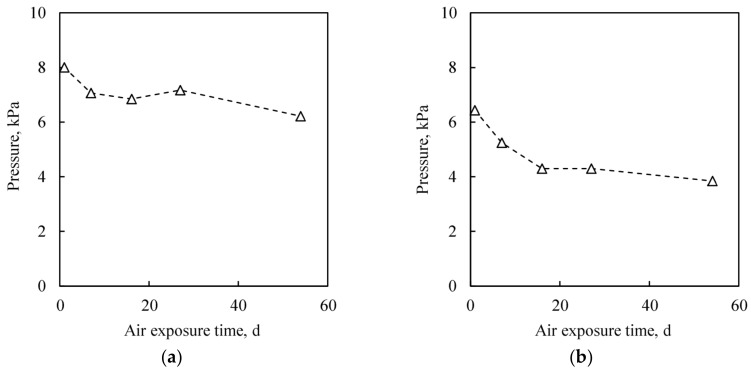
Cavity pressure change with air exposure time. (**a**) Citric acid treated wafer, which was bonded at 350 °C. (**b**) Atomic hydrogen treated wafer, which was bonded at 300 °C.

Figure 8 shows change of the cavities pressure with storage time. The cavity pressure is decreased in the initial 20 days on both citric acid pre-treated sample and atomic hydrogen pre-treated sample. It is considered that the residual gas molecules are re-adsorbed on the Cu surfaces. The coverage of the re-adsorbed molecules on the cavity inside wall of the citric acid and atomic hydrogen pre-treated samples are a 0.9 and 1.7 mono layer, respectively, which are in a reasonable range of typical gas adsorption on a Cu surface.

Estimated leak rate in the worst case by considering the detection error of the zero-balance method (several hundred Pa) is lower than 10^−14^ Pa m^3^ s^−1^ order, which is sufficiently low compared to the reported value by Au-Au bonding [9], and is at least six orders of magnitude lower than that in the rejection limit for military and aerospace electronic systems use defined by the MIL-STD-883G specification of 5.0 × 10^−9^ (Pa·m^3^)/s [34].

### 3.5. AES Measurements: Observation of Cu Re-Oxidation in Atmosphere

**Figure 9:**
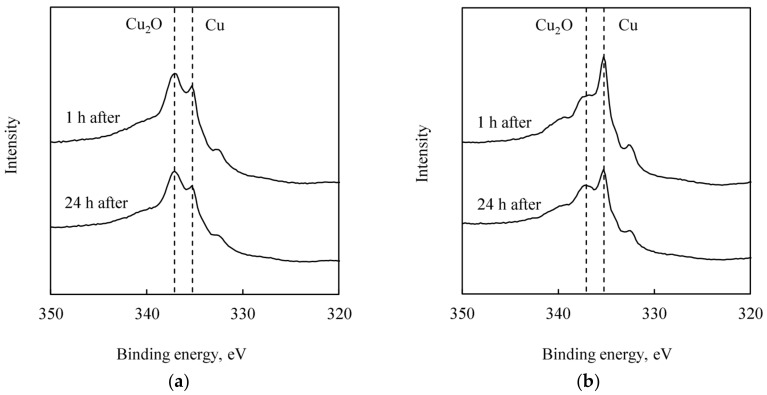
AES spectra of the treated Cu films after exposed to the air for 1 or 24 h. (**a**) Citric acid treated Cu film. (**b**) Atomic hydrogen treated Cu film.

AES spectra of the treated Cu surfaces are shown in Figure 9. The AES peaks at 336.8 eV and 334.9 eV are identified as the binding energy of Cu_2_O and metal Cu, respectively [35,36]. Figure 9a shows that the Cu_2_O peak is already dominant after 1 h exposure to air after the citric acid treatment. In contrast, the metal Cu peak is still dominant after 24 h from the atomic hydrogen treatment, as shown in Figure 9b. These results indicate that the role of atomic hydrogen treatment is not only surface oxide removal but also the prevention of oxide growth on Cu surface in the air.

### 3.6. TDS Measurements: Observation of Chemisorbed Hydrogen Atoms on Cu Surface

**Figure 10:**
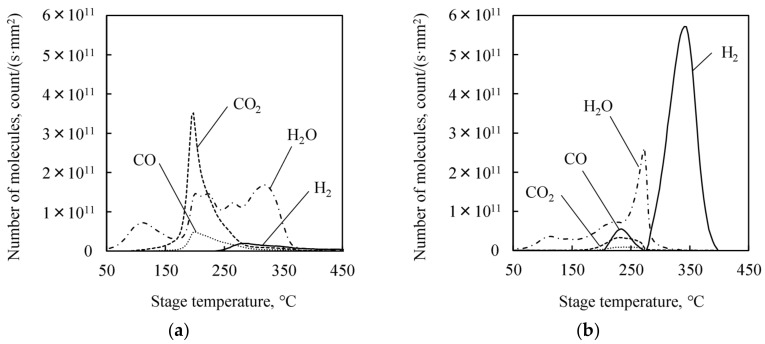
TDS spectra of the treated Cu films after exposed to the atmosphere for 1 h. (**a**) Citric acid treated Cu film. (**b**) Atomic hydrogen treated Cu film.

The spectrum of thermally desorbed molecules from the pretreated Cu films are shown in Figure 10. Most of the desorbed gas species from the citric acid-treated Cu films are H_2_O, CO, and CO_2_, as shown in Figure 10a. These gas species are typical adsorption molecules on Cu surface. On the other hand, for the atomic hydrogen-treated Cu films, the amount of typical desorbed gas is smaller than that from that of the citric acid-treated one. However, a large amount of H_2_ gas is desorbed as shown in Figure 10b. The desorbed H_2_ gas may be generated from the chemisorbed hydrogen which is directly formed from the atomic hydrogen irradiation [37].

**Figure 11:**
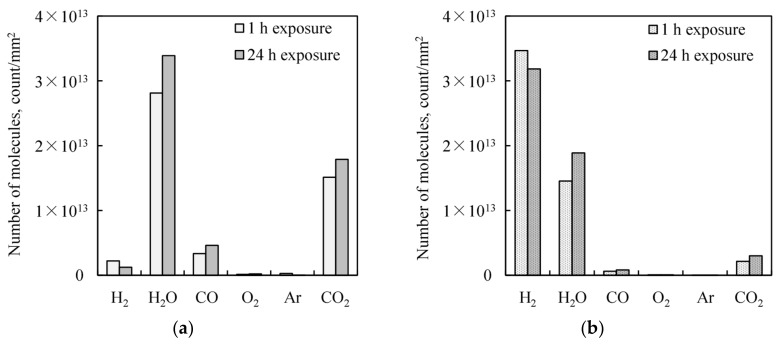
Total amounts of desorbed molecules in the TDS measurements. The treated Cu films are exposed to the atmosphere for 1 or 24 h. (**a**) Citric acid treated Cu film. (**b**) Atomic hydrogen treated Cu film.

Figure 11 shows the amount of the desorbed molecules by integrating TDS peaks. The atomic hydrogen-treated Cu film is occupied with chemisorbed hydrogen even after 24 h storage in the air, as shown in Figure 11a, while chemisorbed hydrogen is not dominant on the citric acid-treated Cu film, as shown in Figure 11b. These results indicate that the chemisorbed hydrogen on the atomic hydrogen-treated Cu surface is stable even in the air, and suppresses other chemical species adsorption, not only oxide, but also carbon species.

### 3.7. Reasons for Low-temperature Vacuum Seal Capability after Storage in Atmospheric Condition

The AES and TDS results indicate that the atomic hydrogen pre-treatment removes Cu native oxide and then forms hydride-like layers such as surface hydrogen or hydroxyl group, which suppress re-oxidation on the Cu surfaces during the wafer storage in atmospheric condition. The hydride-like layer growth on the Cu surface by hydrogen plasma treatment has been reported [21,22,38], and such surface hydrogen species can also suppress the re-oxidation of the Cu surfaces [38]. Such re-oxidation suppression by the chemisorbed hydrogen may enable vacuum sealing at far lower temperature by the atomic hydrogen pre-treatment in comparison with the conventional wet oxide reduction. Moreover, low pre-treatment temperature below Cu re-crystallization point does not promote the coalescence of Cu fine grains, which should proceed during the bonding process instead of the pre-treatment process. Interfacial gap/void filling by the Cu coalescence also plays a key role during low-temperature wafer bonding [16].

It should be noted that the Cu hydride-like layer does not need to be removed by an additional process before bonding, while the surface protection layer such as Cu_3_N or SAM must be removed by thermal treatment before bonding [22,39]. It is considered that desorbed hydrogen atoms can diffuse into the Cu crystals due to their small atomic radius. However, there is a possibility that the strength of the atomic hydrogen-treated wafer is deteriorated due to hydrogen embrittlement. In fact, the shear strengths of the atomic hydrogen-treated wafers were 26–36% lower than the citric acid-treated ones, as shown in Figure 6.

## 4. Conclusions

Wafer-level vacuum sealing by Cu-Cu thermo-compression bonding was achieved at 300 °C by atomic hydrogen pre-treatment for Cu native oxide reduction using a remote-type hot-wire method, while 350 °C of Cu-Cu bonding temperature for vacuum sealing was needed by conventional Cu native oxide reduction using citric acid treatment. The remote-type tool prevents substrate heating by radiation from a hot wire during atomic hydrogen irradiation. As a result, substrate temperature is suppressed below 100 °C, which is lower than re-crystallization temperature of Cu nano-crystal. The removal of Cu native oxide and the suppression of re-oxidation during the storage of a substrate in the air due to chemisorbed hydrogen species on the Cu surfaces are explained by AES and TDS measurements. Vacuum sealing yield, pressure in the sealed cavity, leak rate and bonding strength by Cu-Cu bonding using the atomic hydrogen pre-treatment are almost equivalent to conventional Cu-Cu bonding at higher temperatures using wet oxide removal procedures. This technology will contribute to further low-temperature and low-damage Cu-Cu bonding for hermetic packaging.

## Figures and Tables

**Table 1:** Pre-treatment conditions.

Pre-Treatment	Treatment Temperature	Treatment Time	Remarks
Citric acid solution	25 °C	3 min	After 1 wt % citric acid solution in water, the wafers were rinsed by DI water and ethanol.
Atomic Hydrogen Irradiation	100 °C	10 min	H_2_ flow rate and gas pressure were 500 sccm and 130 Pa, respectively. The hot-wire was heated at 2000 °C.

**Table 2:** Cu-Cu bonding conditions.

Bonding Temperature	Bonding Duration	Bonding Pressure	Ambient Pressure
250, 300, 350 °C	40 min	70 MPa	1.0 × 10^−2^ Pa
